# Low-Intensity Focused Ultrasound-Augmented Multifunctional Nanoparticles for Integrating Ultrasound Imaging and Synergistic Therapy of Metastatic Breast Cancer

**DOI:** 10.1186/s11671-021-03532-z

**Published:** 2021-04-29

**Authors:** Qian Zhang, Wen Wang, Hongyuan Shen, Hongyu Tao, Yating Wu, Liyuan Ma, Guangfei Yang, Ruijiao Chang, Jiaxing Wang, Hanfei Zhang, Chenyu Wang, Furong Zhang, Jiaojiao Qi, Chengrong Mi

**Affiliations:** 1 Department of Ultrasound, General Hospital of Ningxia Medical University, Yinchuan, China; 2 School of Clinical Medicine, Ningxia Medical University, Yinchuan, China

**Keywords:** Nanoparticle, Sonodynamic therapy, Contrast-enhanced ultrasound, Anti-metastasis, LIFU

## Abstract

The metastasis of breast cancer is believed to have a negative effect on its prognosis. Benefiting from the remarkable deep-penetrating and noninvasive characteristics, sonodynamic therapy (SDT) demonstrates a whole series of potential leading to cancer treatment. To relieve the limitation of monotherapy, a multifunctional nanoplatform has been explored to realize the synergistic treatment efficiency. Herein, we establish a novel multifunctional nano-system which encapsulates chlorin e6 (Ce6, for SDT), perfluoropentane (PFP, for ultrasound imaging), and docetaxel (DTX, for chemotherapy) in a well-designed PLGA core–shell structure. The synergistic Ce6/PFP/DTX/PLGA nanoparticles (CPDP NPs) featured with excellent biocompatibility and stability primarily enable its further application. Upon low-intensity focused ultrasound (LIFU) irradiation, the enhanced ultrasound imaging could be revealed both in vitro and in vivo. More importantly, combined with LIFU, the nanoparticles exhibit intriguing antitumor capability through Ce6-induced cytotoxic reactive oxygen species as well as DTX releasing to generate a concerted therapeutic efficiency. Furthermore, this treating strategy actives a strong anti-metastasis capability by which lung metastatic nodules have been significantly reduced. The results indicate that the SDT-oriented nanoplatform combined with chemotherapy could be provided as a promising approach in elevating effective synergistic therapy and suppressing lung metastasis of breast cancer.

## Introduction

Breast cancer has haunted women for years as one of the most threatening malignant tumors. Due to the high heterogeneity and high metastatic ability, it is reported that the distant metastasis of breast cancer accounted for more than 90% of its mortality, whereas the 5-year survival rate of advanced or metastasized patients is only 26%, resulting in a poor clinical outcome [1–3]. The negative characterization of breast cancer has made it hard to be completely cured, promoting the treating strategy to be more challenging in eliminating the primary tumor as well as the distant metastasis.

Traditional therapeutic approaches such as surgery and chemotherapy are still considered to be effective in treating breast cancer [4]. Among all the chemotherapeutic drugs, docetaxel (DTX) is playing a significant role in treating metastatic breast cancer (MBC) and advanced breast cancer (ABC) [5]. As the first-line antitumor drug synthesized by the chemical substances in the yew tree, the anti-tumor effect of DTX is mainly achieved by destroying mitosis and cell proliferation [6]. Owning to its extensive antitumor efficiency, DTX is becoming one of the most effective chemotherapeutic agents in treating breast cancer [7]. However, chemotherapy agent usually induces undesirable effects as well as whole-body toxicity, which have restricted the therapeutic efficiency greatly [8]. Besides, different clinical stages and various personal conditions reveal that a single therapeutic approach may not be efficient enough to meet all the expectations in breast cancer treatment. Hence, there is an urgent need to curb the toxic side effect and enhance treating effectiveness of DTX in the future application requirements.

Ultrasound was first explored in clinical diagnosis due to its extraordinary advantages such as radiation-free, noninvasiveness, and cost-effectiveness [9, 10]. Despite the unique features mentioned above, it has also gained a lot of attention in the therapeutic prospects based on nanostructured materials [11]. Sonodynamic therapy was initially realized by the cavitation and ‘sonoporation effect’ triggered by ultrasound. The generation of ROS during this process induces cytotoxicity toward cancer cells effectively, leading to rapid DNA damage and apoptosis of tumor cells [9]. Unlike the superficial employment of photodynamic therapy (PDT), SDT receives a more pleasant therapeutic outcome in treating deep-seated tumors by low-intensity focused ultrasound (LIFU), which owns merits to prevent undesirable thermal injury during treatment [12, 13]. As one of the essentials in SDT, sonosensitizers have been explored in various tumor therapies [14]. Chlorine e6 (Ce6) was originally employed as a common photosensitizer, but its desirable therapeutic capability and excellent affinition to tumor tissue have made it capable of applying as a sonosensitizer as well [15]. Played as a second generation of chlorine family, Ce6 has aroused an extensive attention in tumor treatment for its desirable ROS generation [16]. However, the single application of Ce6 has constantly aroused instability as well as undesirable skin toxicity, which have all limited the extensive SDT exploration.

In recent years, the development of nanotechnology has played a very important role in many fields, including biosensing, environmental pollution, and contaminant degradation [17–23]. Nanotechnology-based nanoparticles have many advantages, such as smaller diameter, larger external surface area, and lower internal diffusion resistance [24]. Previously, nanoparticles have been involved in the application of various materials such as MOF [25], titanium dioxide [26], and graphene [21]. The rapidly growing trend of nanotechnology combined with cancer treatment has also been explored broadly, paving a feasible way in promoting a combined therapeutic strategy [27]. To realize effective tumor therapeutic applications, the delicately designed nanoparticles are set primarily to improve the toxic chemotherapy agent transportation efficiency via the enhanced permeability and retention (EPR) effect, enabling an increased accumulation in the tumor site [28, 29]. Additionally, the unwanted side effect could also be decreased through encapsulation of chemotherapy. It has been reported that perfluoropentane (PFP) owns an outstanding capacity of transforming from liquid phase to gas phase under irradiation, which could be applied as a novel ultrasound molecular probe, especially in ultrasonic imaging and treatment [30]. More importantly, the initial liquid phase enables PFP to be readily encapsulated in various materials [31]. Besides, the encapsulation of PFP can greatly improve ultrasound imaging capability at tumor site according to the EPR effect mentioned above and avoid size limitation aroused by larger micrometer-sized microbubbles. Except for tumor imaging, the combination of multiple therapeutic approaches is of enormous value in nanosystem-oriented cancer treatment. Specifically, practices including the integration of sonodynamic therapy and chemotherapy have put emphasis to revolutionize the traditional therapeutic efficiencies. Xu et al. [32] demonstrated that due to SDT, an enhanced therapeutic result could be revealed with chemotherapy drugs and thus led to the activation of mitochondria-targeted tumor cell apoptosis. This pattern unveils the synergistic treatment, which is highly concerned in future utilization.

Herein, with the inspirations mentioned above, we intent to exert the all-in-one nanoparticles (CPDP NPs) to establish a diagnostic and therapeutic system, which is realized by the SDT-oriented synergistic therapy combined with chemotherapy, as well as the enhanced ultrasound imaging. Owning to the excellent safety and ideal metabolic stability as a desirable nanocarrier, PLGA has made it favorable in exploring various antitumor capabilities [33, 34]. Hence in this strategy, using PLGA as the outside layer material, PFP could significantly enhance ultrasound imaging through its phase-shift capability triggered by LIFU, while Ce6, a desirable second-generation sonosensitizer, could also be exposed to LIFU to induce ROS generation. Additionally, accompanied by DTX releasing, both chemotherapy and SDT will be realized to achieve a synergistic therapy ultimately. Importantly, the core–shell structure of CPDP NPs can be administrated steadily without hurting normal tissues or cells and also enables nanoparticles to have a relatively higher encapsulation efficiency [35]. Moreover, contents could be well protected in this core–shell structure [36–38], especially for PFP, which can be effectively transformed from liquid to gas due to the structure existence. Using this core–shell structure, the synergistic strategy could be simultaneously demonstrated in a more stable way. Firstly, the synergistic strategy helps to significantly reduce the side effect of DTX by effective encapsulation, which is of great importance to relieve the suffering in treating malignant and aggressive tumors; secondly, compared with single chemotherapy, the combination of SDT and chemotherapy has been verified to be a promising strategy to strengthen therapeutic efficiency. Thirdly, the enhanced ultrasound imaging realized by PFP has optimized the diagnostic strategy and also helped to verify the antitumor effectiveness. Last but not the least, the whole system is safe and stable with excellent biocompatibility. It is highlighted that in this strategy, lung metastasis as well as tumor growth have been remarkably inhibited both in vitro and in vivo. In conclusion, the synergistic strategy demonstrated a productive treatment efficacy against breast malignant tumor and its distant metastasis, and combined with its ultrasound imaging capability, it might become the promising treating strategy in further clinical application.

## Materials and Methods

### Materials

PLGA-COOH (Mw 12,000 Da) was purchased from Jinan Daigang Biomaterial Co., Ltd (Jinan, China). Perfluoropentane (PFP) and agarose were obtained from Sigma-Aldrich Co., Ltd. (St. Louis, MO). Chlorin e6 (Ce6) was purchased from Melone Pharmaceutical Co., Ltd. (Dalian, China). Cell Counting Kit-8 (CCK-8) cytotoxicity assay kit was obtained from Dojindo Molecular Technologies (Tokyo, Japan). 2′,7′-Dichlorodihydrofluorescein diacetate (H2DCFDA) was purchased from MedChemExpress Co., Ltd. (NJ, USA). Propidium iodide (PI) was obtained from Solarbio Science and Technology Co. Ltd. (Beijing, China). Annexin V-FITC/PI was obtained from BD Biosciences (USA). Docetaxel (DTX) was purchased from MedChemExpress Co., Ltd. (NJ, USA). All other reagents were analytical pure products without further purifications. Roswell Park Memorial Institute 1640 medium (DMEM), fetal bovine serum, and tyrisin were purchased from Gibco (ThermoFisher Scientific, USA) and UV spectrophotometer (UV–Vis, Hitachi, Japan).

### Synthesis of CPDP NPs

Ce6-PFP-DTX/PLGA nanoparticles (CPDP NPs) were prepared by a W/O/W double emulsion method according to the previous report [39, 40]. Briefly, 2 mg Ce6 was firstly dissolved in 500 μL methanol. Next, 50 mg of PLGA-COOH and docetaxel (2 mg) were dissolved in 4 mL dichloromethane, and then, the previous solution was simultaneously added into it. Then, 200 μL PFP was added to the above solution. In consequence, the mixture was triggered by an ultrasonic probe (Sonics & Materials Inc., USA) to gain a first emulsion (5 s on and 5 s off, 3 min). To acquire the second emulsion, 8 mL of a poly(vinyl alcohol) (PVA) solution (w/v = 4%) was added into the above emulsion, using the same ultrasonic probe for 2 min. After adding 10 mL 2% isopropyl alcohol into the final emulsion, the solution was mixed under room temperature mechanically for at least 4 h to make dichloromethane volatilized totally. Finally, CPDP NPs were centrifugated for three times (12,000 rpm, 5 min) and then collected and stored at 4 °C for further use. The PDP NPs were prepared in the same way except for Ce6. All experimental processes were operated above ice and conducted strictly in the dark.

### Characterization of CPDP NPs

The particle size and zeta potential of CPDP NPs and PDP NPs were realized by Malvern Zetasizer Nano instrument (Malvern, UK). The morphology was characterized by transmission electron microscopy (TEM) and optical microscopy. To evaluate the stability, CPDP NPs were dissolved in phosphate-buffered solution (PBS) and measured the sizes of 7 days, respectively. Each sample was in triplicate measured. The encapsulation efficiency of CPDP NPs was calculated through the following formula:

Encapsulation efficiency (%) = (Weight of loading DTX or Ce6/Weight of total DTX or Ce6) × 100%. To verify the encapsulation of the various materials, UV–Vis spectra of various samples were investigated (UH5300, Hitachi). The effective encapsulation was also analyzed by TEM.

### Drug-Releasing Rate of CPDP NPs

To evaluate the drug-releasing ability of Ce6 and DTX in CPDP NPs, two solutions with different pHs (phosphate buffer solution, PBS: 7.4, acetate buffer solution, ABS: 5.6) were utilized to test the cumulative releasing efficiency. Briefly, CPDP NPs were firstly dispersed with 1 mL PBS or ABS after the mixture was sealed into a dialysis bag (Mw: 10,000); the whole solution was then transferred into a glass bottle (total volume: 150 mL) in which 149 mL PBS or ABS was added to keep the total solution volume at 150 mL. The glass bottle was then placed into a 37 °C constant temperature shaker, and at different periods (0.5, 1, 2, 4, 8, 12, 24, 48, 72 h), the solution was collected and immediately supplemented with the same volume of medium. Each group was repeated three times. Finally, the concentrations of Ce6 and DTX were measured by Synergy Hybrid Multi-Mode Read (BioTek, USA) at 403 and 229 nm, respectively, and the drug-releasing rate at each time point was calculated.

### In Vitro Ultrasound Imaging

To investigate the ultrasonic capability of CPDP NPs, the emulsion (1 mg/mL) was firstly triggered by a low-intensity focused ultrasound (LIFU) transducer equipment (Ronghai Ultrasonic Medical Engineering Research Center, Chongqing, China), and the conducting pattern was set as 50% duty cycle, 1 s pulse duration under different intensities (1–2 W/cm^2^) for different duration times. For ultrasound imaging, irradiated CPDP NPs were added into the previous prepared agarose model, respectively, using Philips EPIQ5 ultrasound diagnostic instrument (probe frequency: 12 MHz, MI: 0.06) to observe both 2D and CEUS imaging of CPDP NPs. Meanwhile, ImageJ software was applied to analyze the grayscale value of each group.

### Cellular Uptake and In Vitro ROS Generation of CPDP NPs by LIFU Irradiation

The murine breast cancer cell line 4T1 was obtained from Shanghai cell bank of the Chinese Academy of Sciences (Shanghai, China) and incubated in RPMI 1640 medium mixed with 10% FBS and 1% streptomycin/penicillin at 37 °C in a 5% CO_2_ humidified incubator.

4T1 cells were first incubated as previous condition at a density of 1 × 10^4^ cells per dish to test the cellular uptake using CLSM in different time intervals (1 h, 2 h, 4 h, 8 h). To verify the ROS generation, cells were separated into the following 5 groups: control, CPDP NPs, LIFU, Ce6 + LIFU, CPDP NPs + LIFU. After 24-h conventional culture, the medium was replaced by CPDP NPs (200 μL, 0.8 mg/mL) or Ce6 solution (200 μL), respectively, and the cells were co-incubated for another 3 h. Then, LIFU irradiation (2 W/cm^2^, 120 s) was conducted, respectively, according to different groups. After co-incubation and LIFU treatment, 100 μL diluted DCFH-DA solution was added, and each group was cultured in the previous incubator for 15 min. A confocal laser scanning microscope (CLSM) was used to confirm the result of reactive oxygen species production, and the corresponding fluorescence intensities were measured by ImageJ software.

### In Vitro Cytotoxicity and Concerted Treatment Capability of CPDP NPs

A CCK-8 assay was applied to assess the cytotoxicity of CPDP NPs. Briefly, 4T1 murine breast cancer cells were incubated in a 96-well plate, with a density of 1 × 10^4^ per well for 24 h. Then, CPDP NPs were diluted with serum-free RPMI 1640 medium at various concentrations (0,0.2,0.4,0.6,0.8 mg/mL, *n* = 3), with or without LIFU irradiation (2 W/cm^2^, 120 s). After another 24 h of cocultured process, the cell viability of 4T1 cells was performed.

To evaluate cell apoptosis efficiency of concerted treatment, 4T1 cells were cocultured as previous for 24 h and then separated into five following groups: (1) control (without any treatments), (2) LIFU (only with LIFU exposure at 2 W/cm^2^), (3) CPDP NPs (only with CPDP NPs solution at 0.8 mg/mL), (4) PDP NPs + LIFU and (5) CPDP NPs + LIFU. After various nanoparticles coincubation (200 μL) and LIFU exposure, each group was treated with annexin V (5 μL) and propidium iodide (5 μL) double staining for 20 min and analyzed through a flow cytometry protocol.

### In Vitro Inhibition of Cell Metastasis

To investigate the inhibition of cell metastatic ability, wound healing assay and transwell assay were designed. For wound healing assay, 4T1 cells were conventionally cultured as previous in the 6-well plate. After cell growth to an 80% confluency, a pipette tip (10 μL) was applied to conduct a manmade scratch along the center of the 6-well plate. Then, cells were treated in the same groups mentioned above. After a continuously coincubation for 24 h, the cells were washed with PBS 3 times and observed under optical microscopy (Olympus, Japan).

For transwell assay, the top compartment of the transwell chamber (Corning, San Diego, USA) was primarily applied to imitate the extracellular matrix in vivo. 4T1 cells at a density of 1 × 10^5^ cells per well were seeded into the upper chamber in a serum-free RPMI 1640 medium, while the bottom compartment was filled with a complete culture medium mixed with 10% FBS. Then, cells were separated as the same as above groups and treated for 24 h, respectively. After that, cells in the bottom surface were fixed with paraformaldehyde and stained with crystal violet. The results were observed with light microscopy (Olympus, Japan).

### In Vivo Ultrasound Imaging

Healthy female BALB/c mice (4 weeks) and Kunming mice (4 weeks) were obtained from Ningxia Medical University Laboratory Animal Center. All animal experiments were conducted under the guideline approved by the Animal Welfare Ethics Review Committee of Ningxia Medical University. To establish the mice tumor-bearing model, BALB/c mice were inoculated with 4T1 breast cancer cells (1 × 10^7^/mL) at the right flank. After tumor size grew to 60–80 mm^3^, BALB/c mice were injected with CPDP NPs (200 μL, 1 mg/mL) through the tail vein intravenously. 24 h later, tumor sites of the mice were conducted with LIFU (2 W/cm^2^, 120 s), and then, the 2D and CEUS imaging were acquired through the Philips EPIQ5 ultrasound diagnostic instrument mentioned previously. The grayscale analysis was measured by ImageJ software.

### In Vivo Synergistic Therapeutic Efficiency of CPDP NPs

After 4T1 cancer cells inoculation, the size of the tumor was recorded every two days, and the volume of the tumor was calculated by the formula as: Volume = 1/2 × Length × Width^2^. Tumor size and mice body weight were recorded every 2 days, while the pictures of tumor growth were recorded every 3 days. When tumor volume reached 60–80 mm^3^, mice with similar tumor size were randomly divided into the same five groups: control, LIFU, CPDP NPs, PDP + LIFU, and CPDP NPs + LIFU (*n* = 3). Each group was intravenously injected with various NPs (200 μL) via tail vein except for control group (200 μL PBS instead). Twenty-four hours later, the tumor site was exposed with LIFU irradiation (2 W/cm^2^) for 120 s. The whole SDT administration was repeated every 3 days and lasted for 18 days. The body weight and tumor volume of the mice were measured and calculated. After the treatment, mice were killed and tumor tissues were sent to H&E, TUNEL, and PCNA for furtherhistological analysis.

### Biosafety of CPDP NPs In Vivo

To investigate the biosafety of CPDP nanoparticles in vivo, healthy female Kunming mice (*n* = 3) were separated into the following 4 groups: control, 5 mg/mL, 10 mg/mL, and 20 mg/mL. The CPDP NPs (200 μL) were injected through the mice tail vein; then, the mice were free access to food and water without any further administration. The bodyweight of mice was measured every 2 days. After 30 days, mice were killed and the blood samples were collected for blood cell and biochemistry analysis. The major organs (heart, liver, spleen, lung, and kidney) were collected and investigated for H&E staining, respectively.

### In Vivo Inhibition of Lung Metastasis

To evaluate the lung metastasis inhibition of each group, the whole process was conducted by observing the number of metastatic nodules in the lung as well as an H&E staining histology assessment. After all the mice were euthanized, the lung tissues were removed and fixed; then, photographs of cancer nodules were taken and lung tissues were further analyzed with H&E staining.

### Statistical Analysis

Measurement data were all performed 3 times and expressed as mean ± standard deviation (SD) and analyzed by one-way ANOVA or a standard Student’s *t* test through the SPSS software (version:19.0), while *p* value < 0.05 was considered to be statistically significant.

## Results

### Characterization of CPDP NPs and Drug-Releasing Efficiency

**Fig. 1:**
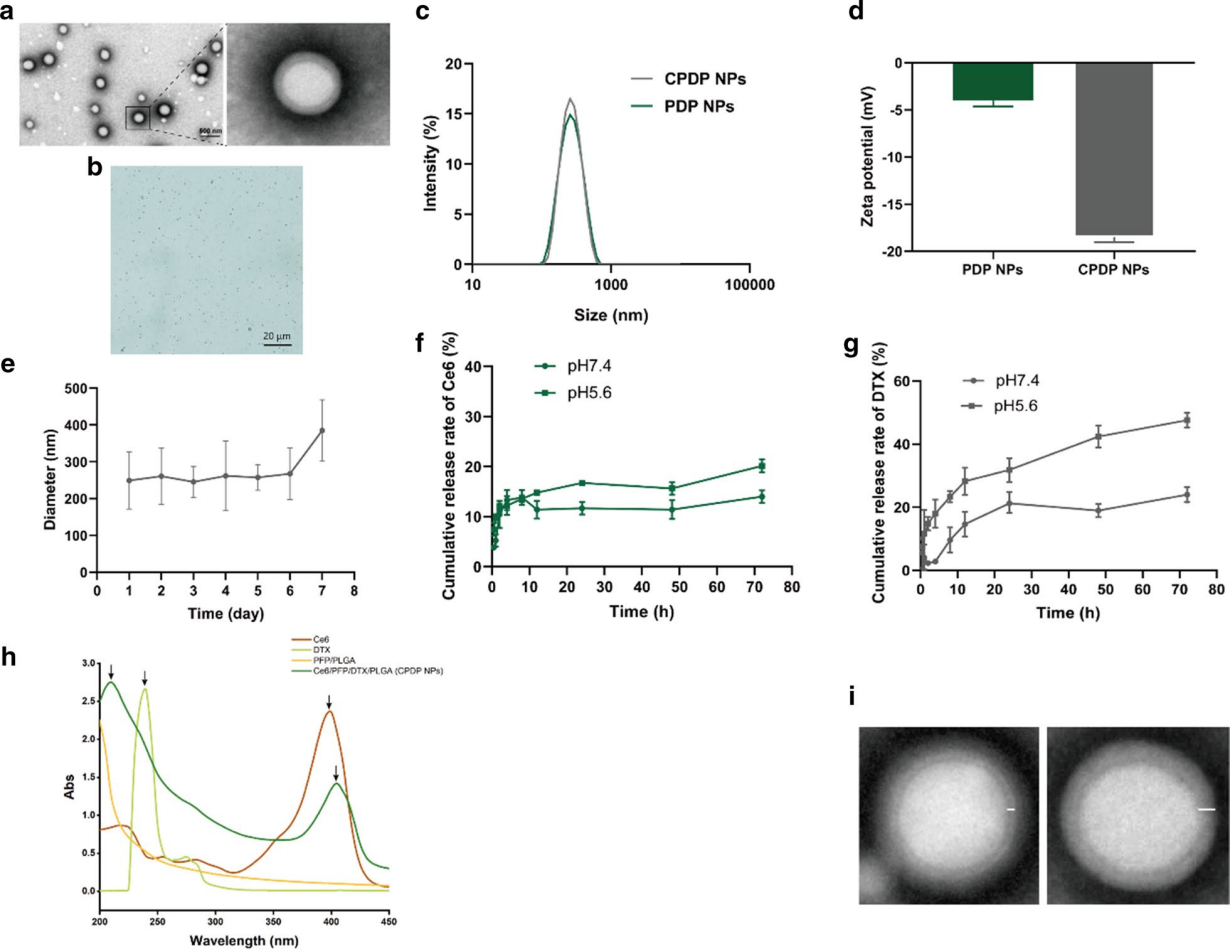
**a** TEM (scale bar: 500 nm) and **b** light microscope image of CPDP NPs (scale bar: 20 μm). **c** Size distribution and **d** zeta potential of PDP NPs and CPDP NPs. **e** The size distribution of CPDP NPs within 7 days. **f** The releasing rate of Ce6 and **g** the releasing rate of DTX under different conditions (pH 7.4 and pH 5.6, *n* = 3). **h** The UV–Vis spectrum of Ce6, DTX, PFP/PLGA and CPDP NPs, respectively. The arrows of CPDP NPs show the characteristic peaks of Ce6 and DTX, indicating the effective encapsulation of both materials. **i** The TEM result of two nanoparticles. The PFP/PLGA nanoparticle exhibits the thin shell and the round core (left). The CPDP nanoparticle encapsulated both Ce6 and DTX shows the much thicker shell and the oval-shaped core (right)

A double emulsion method was applied in fabricating CPDP NPs, which encapsulated both phase-shift material PFP, sonosensitizer Ce6, and chemo-drug DTX simultaneously. When dispersed into PBS or deionized water, the solution presented a light gray appearance. CPDP NPs exhibited a homogeneous spherical shape and a clear core–shell structure, whether being observed via optical microscopy or transmission electron microscopy (Fig. 1a, b). The mean diameters of CPDP NPs and PDP NPs were 249.5 ± 77.46 nm and 246.6 ± 81.01 nm, and the average surface zeta potentials were − 18.47 ± 0.55 mV and − 3.987 ± 0.66 mV, respectively (Fig. 1c, d). The size of CPDP NPs guaranteed that it could be accumulated into the tumor site passively through the EPR effect [40]. The different charge between CPDP NPs and PDP NPs is mainly due to the negatively charged Ce6 [41]. In addition, the negative zeta potential of CPDP NPs indicated a lower plasma protein adsorption, verifying the relative stability of nanoparticles. The particle size distributions were maintained between a range of 249.5 and 385.1 nm in 7 days (Fig. 1e), demonstrating the relative steadiness of CPDP NPs. According to the standard curve, the encapsulation efficiency of DTX and Ce6 was 83.84 ± 1.39% and 60.54 ± 3.79%, respectively.

As the drug-releasing efficiency of Ce6 and DTX from CPDP NPs indicated in Fig. 1f, g, a nearly twofold increase of DTX-releasing index was recorded at pH 5.5 compared with nanoparticles dissolved in pH 7.4, which indicated the reasonable drug-releasing rate of DTX could be achieved effectively in an acid tumor microenvironment. The above results in all exhibited the delicate designed CPDP NPs can exert a steady and timely chemotherapeutic drug release in the acid tumor environment, and also fundamentally desirable to be prepared for SDT.

As the UV–Vis exhibited, DTX and Ce6 revealed unique absorption peaks in 229 nm and 403 nm, respectively, and on the contrary, PFP/PLGA showed none of the peaks. It should be noted that the spectrum of CPDP NPs exhibited the similar peaks both near the above two wavelengths, while the rest showed the same tendency of PFP/PLGA spectrum, indicating the successful encapsulation of the various materials (Fig. 1h). To further verify the effective encapsulation, Fig. 1i indicated PFP/PLGA nanoparticle owns a much thinner shell and the round core, while the CPDP nanoparticle reveals a relatively thicker shell and the oval-shaped core due to the encapsulation of both DTX and Ce6.

### In Vitro Ultrasound Imaging

**Fig. 2:**
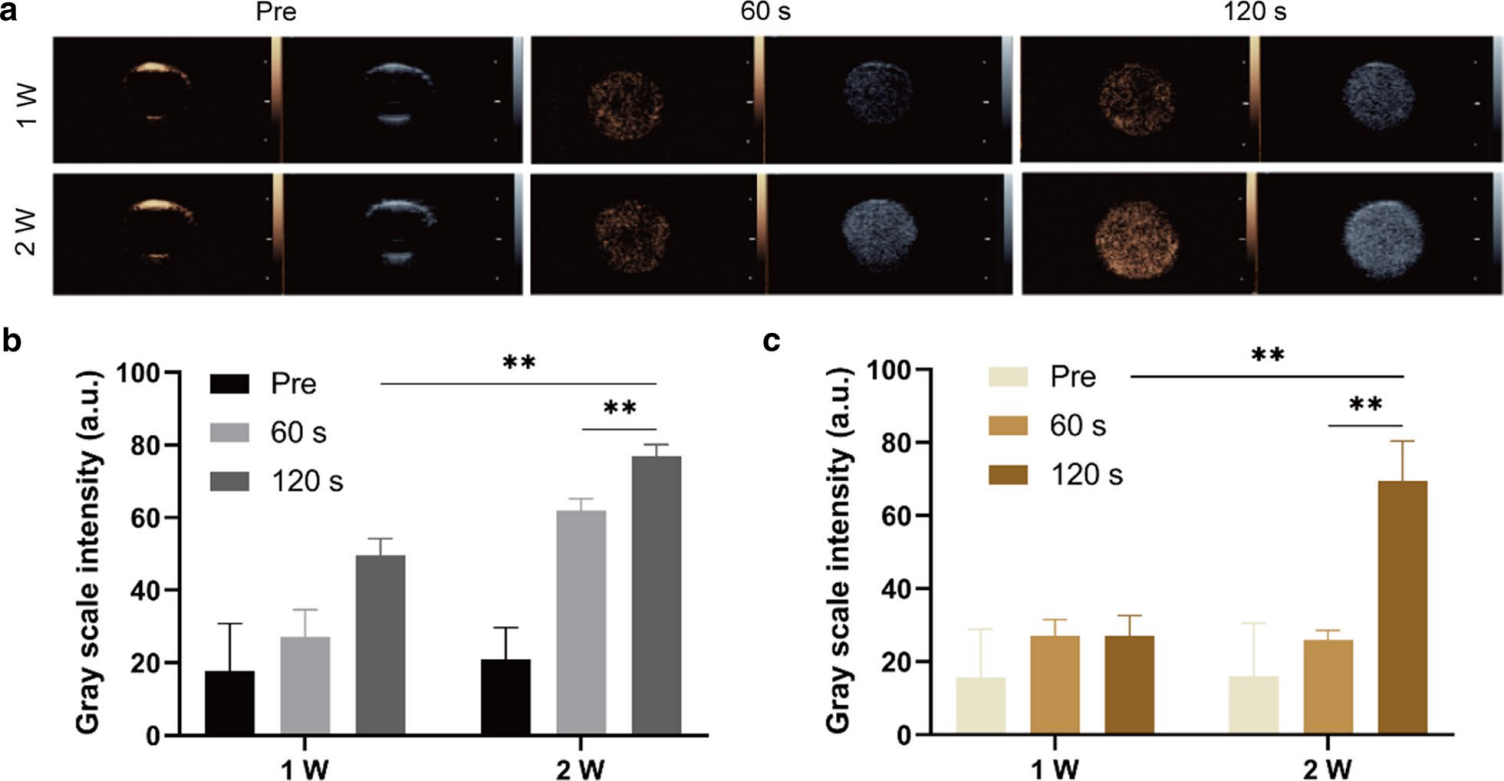
**a** Ultrasound images of both 2D and CEUS under different LIFU intensities and duration times. **b** The corresponding grayscale intensity at different intensities and time of 2D imaging and **c** CEUS imaging (**p < 0.01, *n* = 3)

It is highlighted that PFP owns excellent phase-shift ability. The liquid-to-gas transformation not only helps nanoparticles aggregate within the tumor site but also entitles its capability to enhance the efficiency of ultrasound imaging [42]. To demonstrate that, LIFU irradiation was applied as a trigger to induce the phase transformation of PFP, namely the acoustic droplet vaporization (ADV) effect [43]. The results showed that the grayscale intensities were kept at a relatively low level before LIFU irradiation, while after the intensity and irradiating time of LIFU increased, the tendency of enhanced ultrasound imaging was revealed both in 2D and in CEUS (Fig. 2a). The acoustic analysis of ImageJ further convinced the result by elevated grayscale value (Fig. 2b, c), which was consistent with the imaging findings. It should be noted that the most significant result of 2D and CEUS gained when LIFU intensity achieved 2 W/cm^2^ and lasted for 120 s. The results above demonstrated that PFP was successfully encapsulated in CPDP NPs, and ultrasound imaging capability had been significantly promoted under higher intensity as well as longer time of LIFU administration.

### Cellular Uptake and In Vitro ROS Generation of CPDP NPs by LIFU Irradiation

**Fig. 3:**
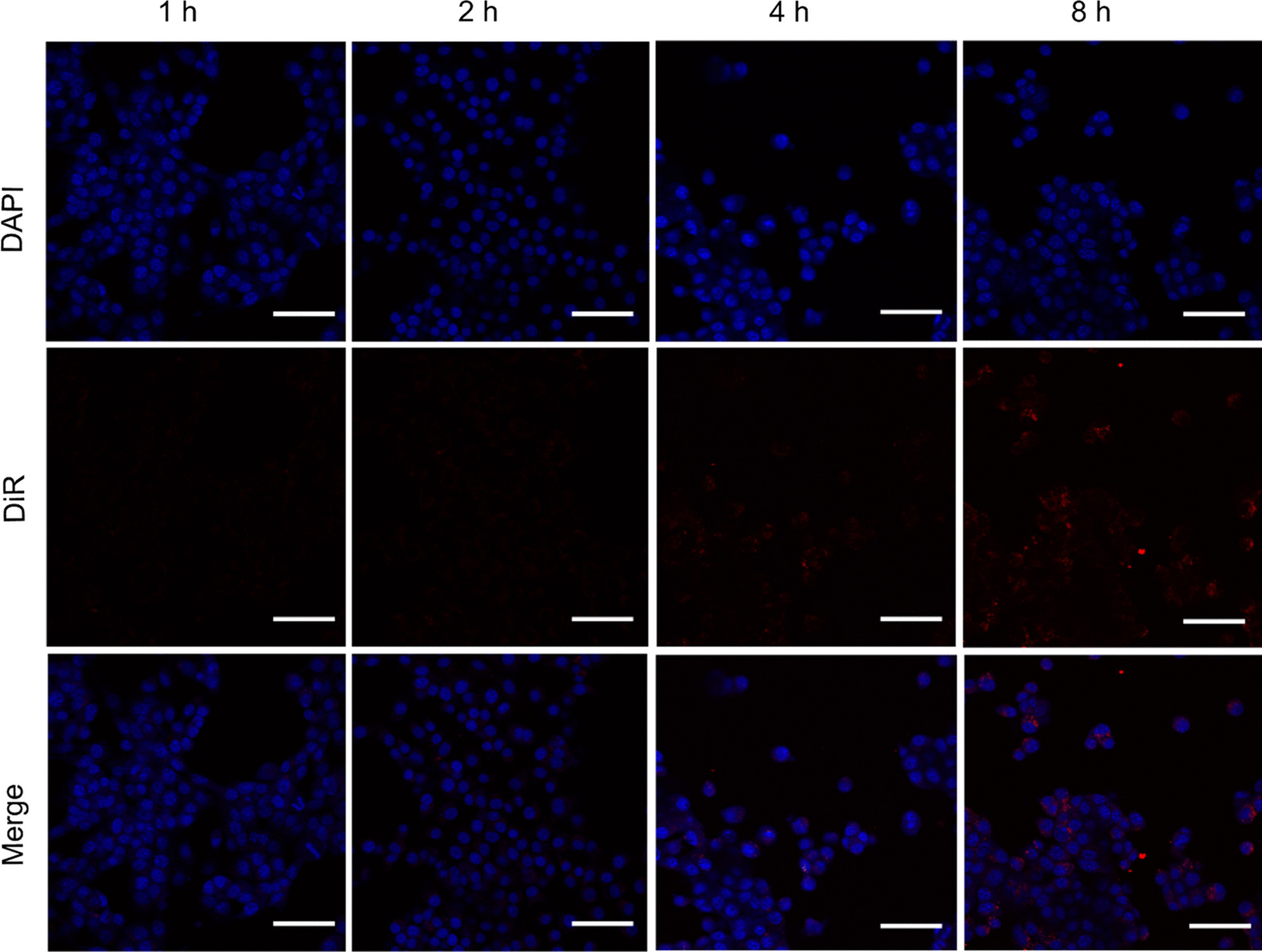
The 4T1 cellular uptake of CPDP NPs during different time intervals (Blue: DAPI stained 4T1 cells. Red: DiR-labeled CPDP NPs, the scale bar is 50 μm)

As the CLSM result showed in Fig. 3, the cellular uptake of CPDP NPs exhibited an enhanced trend in different time intervals, reaching the most significant aggregation at the 8-h coincubation.

**Fig. 4:**
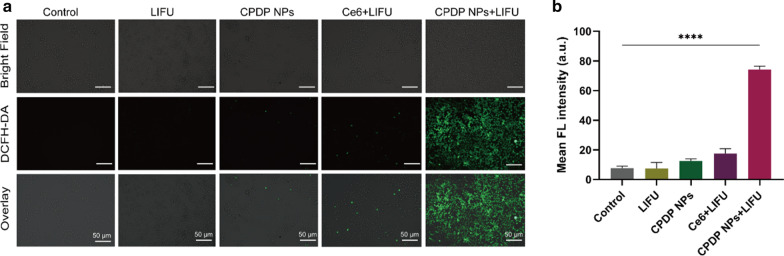
**a** CLSM images of ROS generation with various treatments and **b** the corresponding FL intensity analysis (****p < 0.0001, *n* = 3). The scale bars are 50 μm

The main strategy of sonodynamic therapy (SDT) is the generation of ROS—a series of single-electron reduction products—to induce cancer cell apoptosis and inhibit cell proliferation [44]. It is highlighted that when exposed to ultrasound, the sonosensitizer is prone to trigger ROS production; meanwhile, a considerable amount of energy will be released during the whole process [45]. Given that both ultrasound and sonosensitizer are dispensable elements to promote SDT, hence, the intracellular ROS generation was designed and analyzed to investigate the differences between divided groups. According to Fig. 4a, the amount of ROS generated by the free Ce6 plus LIFU irradiation group was negligible, which may because the rapid metabolism of free Ce6 leads to an unsatisfactory ROS production. On the contrary, the strongest fluorescent intensity was revealed by CPDP NPs + LIFU group. It was assumed that the encapsulated Ce6 was well protected and thus had been kept away from being metabolized. As a consequence, after LIFU stimulation, Ce6 was released to produce abundant ROS. Comparatively, there were no significant fluorescent signals found in other groups (Fig. 4b).

### In Vitro Cytotoxicity and Concerted Treatment Capability of CPDP NPs

**Fig. 5:**
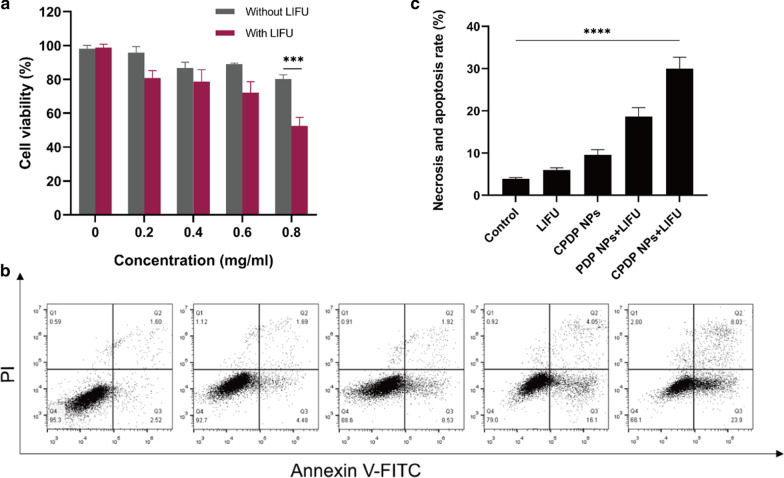
**a** Relative cell viability with or without LIFU irradiation under different CPDP NPs concentrations. **b** 4T1 tumor cell apoptosis and necrosis by flow cytometry assay and **c** the data of corresponding necrosis and apoptosis rate analysis (****p < 0.0001, ***p < 0.001, *n* = 3)

Cell Counting Kit-8 (CCK-8) assay was introduced to test the in vitro cytotoxicity of CPDP NPs. In this respect, different groups were designed with or without LIFU irradiation at different concentrations. The results indicated that after a 24-h co-incubation without LIFU exposure, there was no obvious effect of the survival rate of CPDP NPs even at the highest concentration (0.8 mg/mL), demonstrating the desirable biosafety of CPDP NPs (Fig. 5a). By contrast, it showed that there was a striking decrease of cell viability after LIFU irradiation, showing the combination of CPDP NPs and LIFU has remarkably triggered 4T1 cell death, which was consistent with the in vitro ROS generation.

To further evaluate SDT efficacy, a flow cytometry assay was introduced. As the results shown in Fig. 5b, c, the index of cell necrosis and apoptosis was highest observed in CPDP NPs + LIFU group, while other groups showed no obvious and necrosis and apoptosis. Notably, the necrosis and apoptosis rate of CPDP NPs + LIFU group was threefold higher than that of CPDP NPs only group, which ensured the significant tumor cell death efficiency of SDT from another respect. Intriguingly, compared with PDP NPs + LIFU group, cell necrosis and apoptosis rate in CPDP NPs + LIFU group was significantly increased, exhibiting the synergistic therapy efficiency of SDT and chemotherapy.

### In Vitro Inhibition of Cell Metastasis

**Fig. 6:**
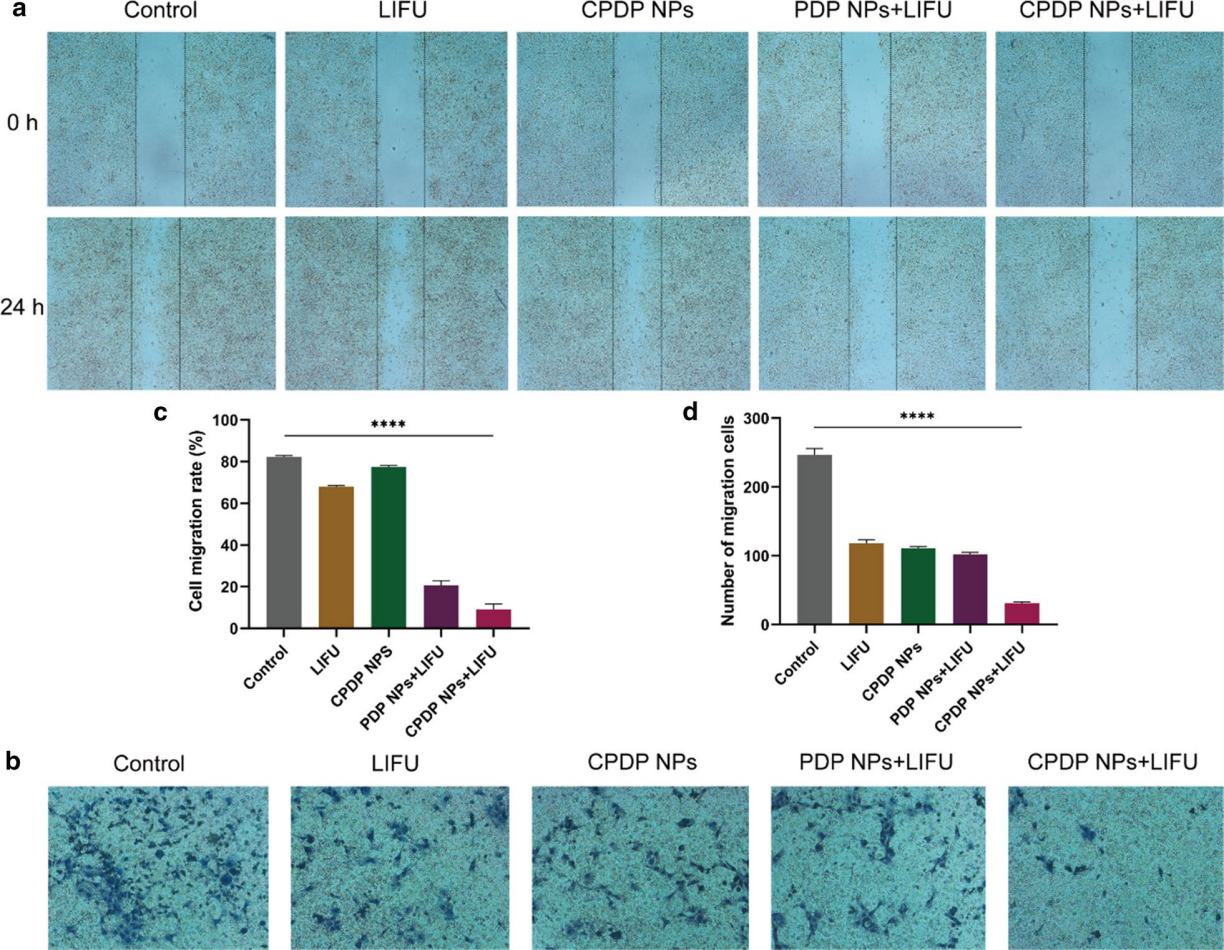
**a** The wound healing and **b** The transwell assay after various treatments. **c** The corresponding migration rate of wound-healing assay. **d** The corresponding migration number of tranwell assay (****p < 0.0001, *n* = 3)

The invasive and migration capability of tumor cells are indispensable in tumor progression [46, 47]. As shown in Fig. 6a, the closure between the physical gap of CPDP NPs + LIFU group was significantly wider than other groups, indicating a relatively slower speed of migrating efficiency. According to the ImageJ software analysis (Fig. 6c), the migration rate of CPDP NPs + LIFU group was also remarkably reduced compared with other groups.

Similarly in the transwell assay, compared with the swift migration speed of other groups, CPDP NPs + LIFU group revealed a significant reduction of cell number (Fig. 6b), which demonstrated an excellent anti-migration capability of the synergistic therapy. Specifically, with the absence of SDT (CPDP NPs only and LIFU only group), the number of tumor cells was mildly decreased (Fig. 6d). On the whole, due to the combination of SDT as well as chemotherapy, metastasis of 4T1 cells has been remarkably inhibited in vitro.

### In Vivo Ultrasound Imaging

**Fig. 7:**
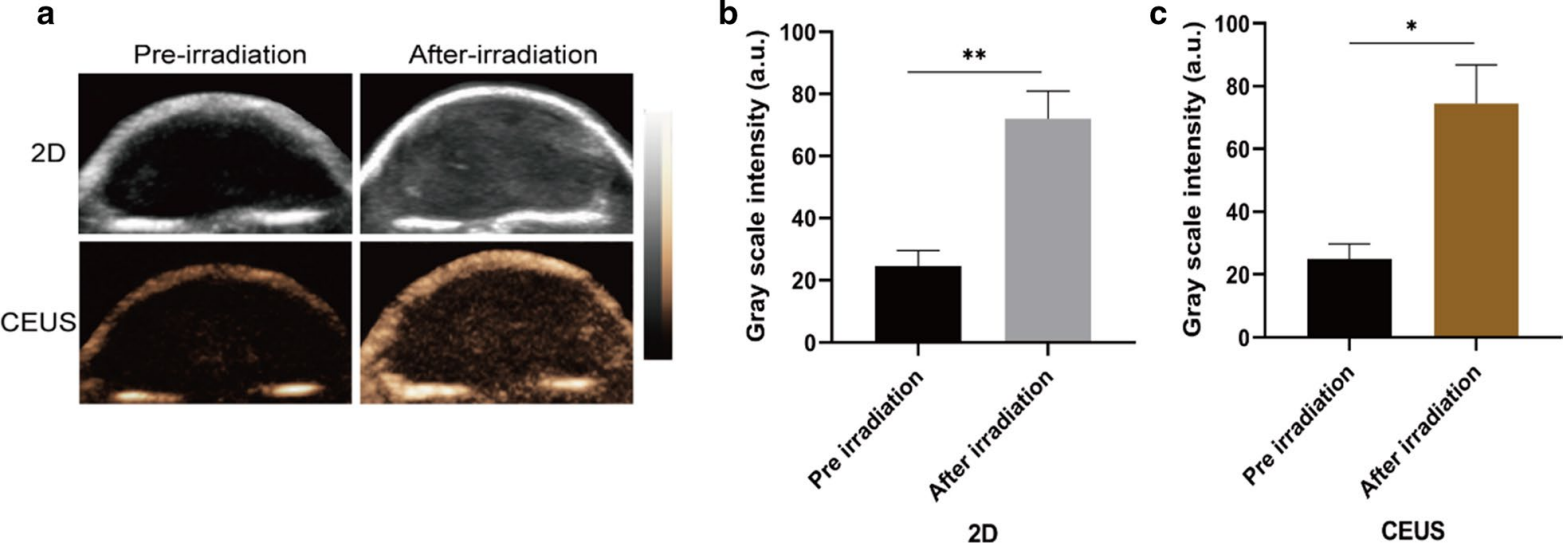
**a** 2D and CEUS images with and without LIFU irradiation. **b**, **c** The corresponding grayscale intensity analysis measured by ImageJ (**p < 0.01, *p < 0.05, *n* = 3)

Since PFP was encapsulated in CPDP NPs, it is also necessary to evaluate the characteristic ultrasound imaging capability in vivo. After the injection via the tail vein of CPDP NPs, LIFU was then applied to the tumor site to acquire both 2D and CEUS imaging (Fig. 7a). The clear graphic difference between the two groups indicated that after LIFU irradiation, the corresponding intensity of CPDP NPs was elevated obviously compared with the pre-irradiation group. Further data of the average echo intensity also confirmed this result, which was also consistent with the in vitro imaging result previously (Fig. 7b, c).

### In Vivo Synergistic Therapeutic Efficiency of CPDP NPs

**Fig. 8:**
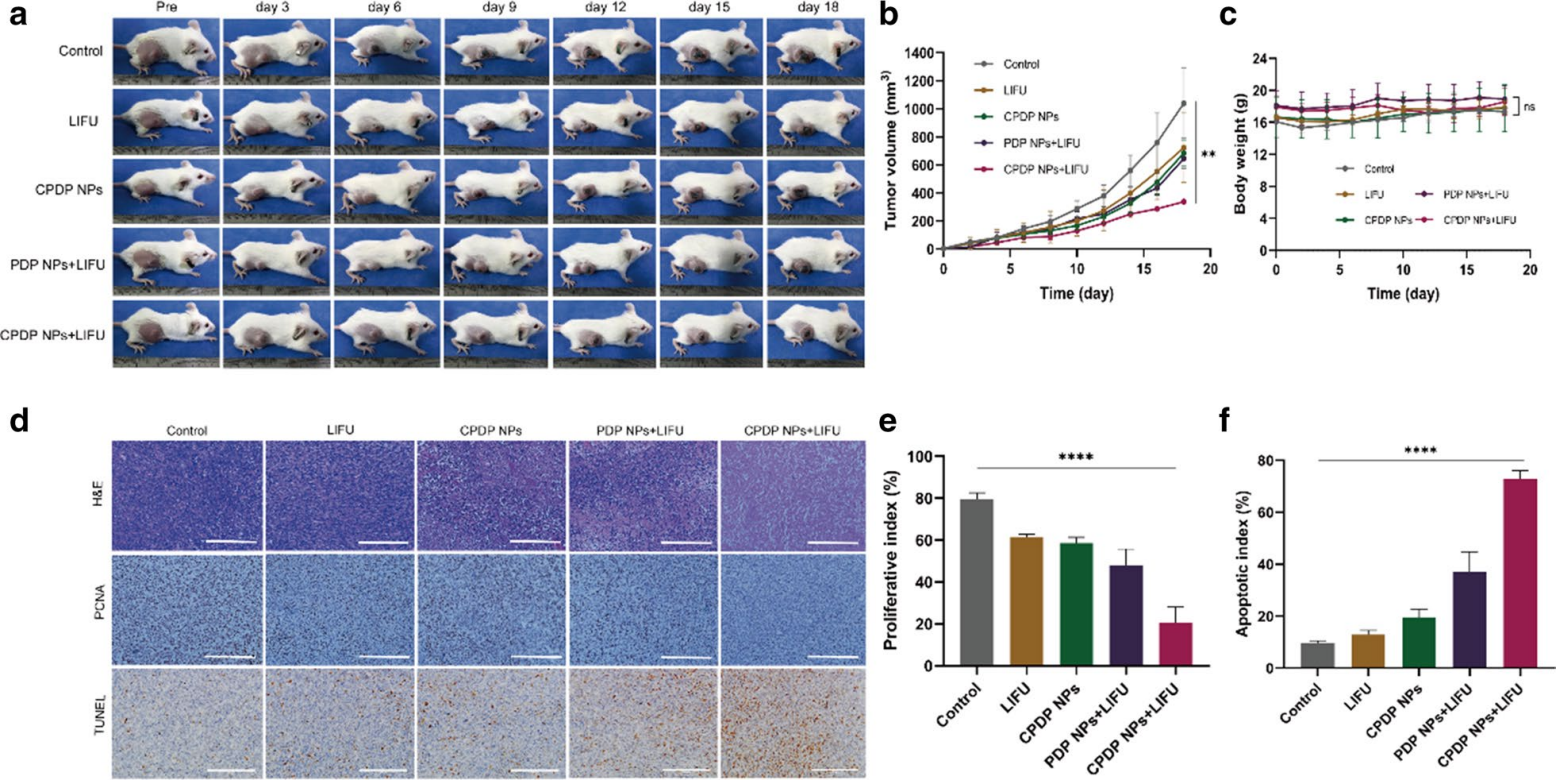
**a** Images of tumor-bearing mice under various treatments within the certain 18 days (*n* = 3). **b** Tumor volume analysis according to various treatments (**p < 0.01). **c** Weights of tumor-bearing mice under various treatments (*ns* no significance, *n* = 3). **d** H&E, PCNA and TUNEL results under various treatments (scale bar: 200 μm). **e** Analysis of PCNA proliferation index of tumors under various treatments. f Analysis of TUNEL apoptotic index of tumors under various treatments (****p < 0.0001, *n* = 3)

Seeing from Fig. 8a, b, the tumor volume of CPDP NPs + LIFU group was significantly smaller after 18 days of treatment than that of other groups, which may attribute to the effectiveness of ROS originated from SDT treatment as well as chemotherapy to exert a valid synergistic therapy efficiency. Similarly, photographs of mice-bearing tumors (Fig. 8a) also showed the same trend, verifying the cooperative treating efficacy of CPDP NPs triggered by LIFU exposure. Furthermore, there was no obvious weight reduction of mice between different groups (Fig. 8c). The results above all indicated a much higher inhibition rate of CPDP NPs + LIFU group, revealing that the synergistic therapy could significantly prevent tumor growth.

To further testify the therapeutic results of all groups, both H&E, PCNA, and TUNEL staining were utilized (Fig. 8d). The proliferate rate of PCNA in CPDP NPs + LIFU group was only 20.50%, which was fourfold lower than control group, threefold lower than LIFU and CPDP NPs only group and twofold lower than PDP + LIFU group, respectively, demonstrating a significant anti-tumor proliferation rate (Fig. 8e). As it is shown in Fig. 8d, f, the TUNEL results indicated CPDP NPs + LIFU group exhibited an obvious apoptosis index of 72.86%, which was much higher than control (9.66%), LIFU (12.86%), CPDP NPs (19.59%), and PDP NPs + LIFU (37.06%) group. The results above all demonstrated the effectiveness of synergistic therapy exerted in vivo, which was also proved consistent with the previous in vitro results.

### Biosafety of CPDP NPs In Vivo

**Fig. 9:**
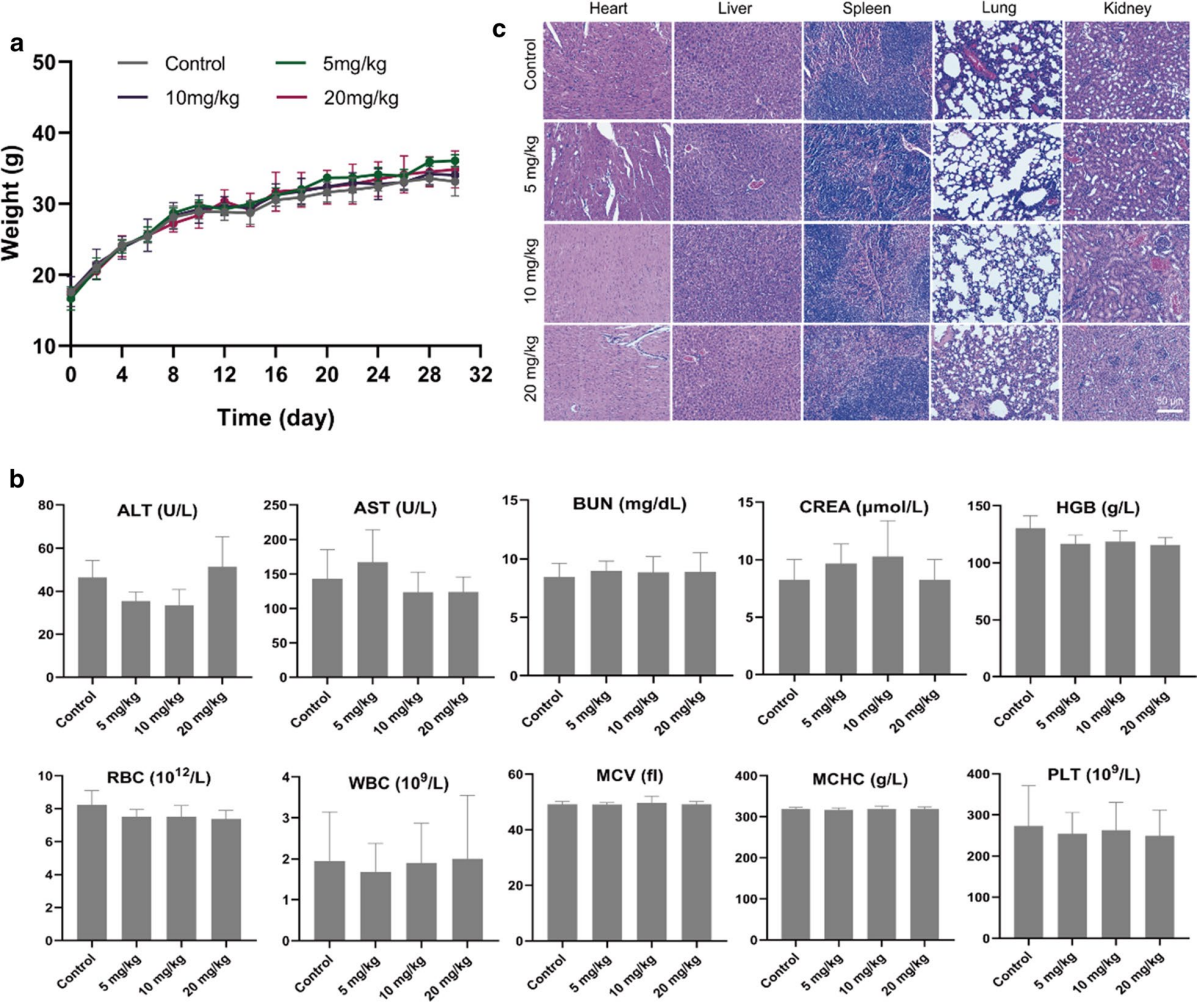
**a** The weights of healthy Kunming mice under various concentrations of CPDP NPs (*n* = 3). **b** The blood biochemistry and blood routine examination under various concentrations of CPDP NPs within a certain period of 30 days (*n* = 3). **c** H&E results of different organs (heart, liver, spleen, lung, and kidney) of mice under the same treatment (scale bar: 50 μm)

Despite the effective therapeutic outcome, it is of great importance to explore the biosafety of the novel established nanoparticles as well. On behalf of the safe distribution of CPDP NPs in vivo, the metabolic safety was conducted. The results showed that instead of apparent body weight loss, the mice body weight elevated gradually in all the groups of mice (Fig. 9a), which indicated a negligible negative influence of CPDP NPs. In addition, as various organs and the blood samples exhibited in Fig. 9b, no significant changes were observed in blood cell, biochemistry analysis index, and H&E staining (Fig. 9c) among different treating groups, indicating the excellent biosafety of CPDP NPs in vivo.

### In vivo Inhibition of Lung Metastasis

**Fig. 10:**
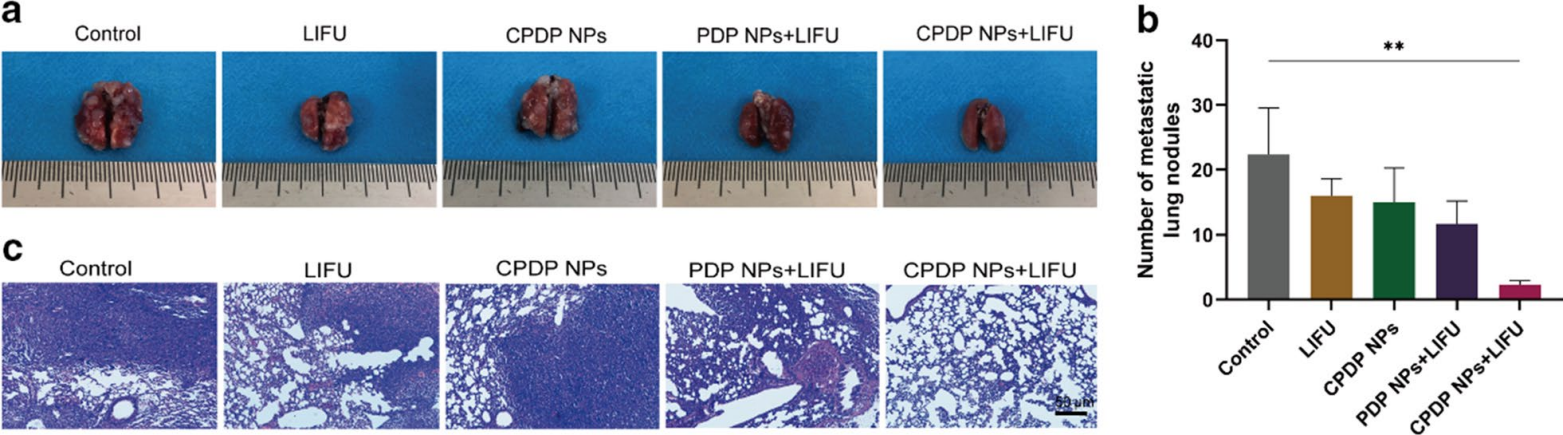
**a** Images of the general appearances of lung tissues. **b** The analysis of the metastatic lung nodules between various treatments (**p < 0.01, *n* = 3). **c** Corresponding images of lung metastatic H&E staining results (scale bar: 50 μm)

It is well established that the lung is the main target organ for distant metastasis of breast cancer [48]. In order to evaluate the suppressing efficiency of metastasis, lung tissues of mice were utilized for anti-metastatic investigation. As seen from Fig. 10a, b, compared with control, LIFU, CPDP NPs, and PDP + LIFU group, the CPDP NPs + LIFU group exhibited the most remarkable decrease of lung nodules, which suggested its desirable lung metastatic inhibition efficiency. A similar trend of decreasing also further indicated by H&E staining (Fig. 10c), which in all demonstrated this synergistic therapy strategy could exert effective effort in eliminating lung metastasis in mice.

## Discussion

It has been greatly acknowledged that the metastasis of breast cancer will extensively influence its poor prognosis [3, 49]. As a desirable therapeutic approach, SDT may serve for its high efficiency and deep penetrating capability has been extensively convinced [11, 50]. Admittedly, since certain limitations exist in the single application of sonosensitizers, using SDT alone may still be less sufficient in further cancer exploration. The development of nanotechnology combined with clinical medicine has been promoted significantly in recent years, owning to the inspiring merits such as negligible toxicity, none invasiveness, and excellent biocompatibility. Using this novel approach, researchers have made many efforts in exploring multifunctional therapeutic strategies to enhance antitumor efficiency.

The biosafety is the priority of nano-agents. As a widely accepted material approved by the Food and Drug Administration (FDA) certification, it is highlighted that PLGA could be performed as a desirable carrier in application [51, 52]. Based on its advantages, we established a nano-system to realize multifunctional therapy efficiency, exploiting PLGA as the outer structure to encapsulate sonosensitizer Ce6, phase-shift material PFP and chemotherapeutic agent DTX. The CPDP nanoparticles (CPDP NPs) were primarily observed by the core–shell structure and appropriate size so that a desirable aggregation through the EPR effect could be achieved. The cell viability of CPDP NPs has been proved to be above 80% after 24-h coincubation, indicating the well safety of this nanoplatform. Besides, the phase transformation of encapsulated PFP has also guaranteed CPDP NPs as a contrast-enhanced agent when activated by LIFU. The enhanced ultrasound imaging capability will not only ensure the ideal therapeutic window but also promote the promising future for CPDP NPs to realize an integration of accurate diagnosis and precise treatment.

The key strategy in SDT is the generation of ROS. Compared with the single employment of Ce6, the encapsulated Ce6 in PLGA exerted a desirable protection, which could be verified by the ROS result. In our study, there was only a negligible amount of ROS generated by single use of Ce6, while nanoparticle-encapsulated Ce6 produced a considerable amount of ROS, which was further proved by SDT efficiency. The result indicated that ROS generation was preserved by the encapsulated Ce6 in CPDP NPs, which could lay a firm foundation for later tumor inhibition. Besides, the drug-releasing rate showed even at pH 5.5, little Ce6 was released from nanoparticles, demonstrating a well protection of sonosensitizer by PLGA, which was further proved by the ROS production and SDT outcome with high efficiency. Interestingly, the drug-releasing efficiency of DTX and Ce6 at pH 5.5 was quite different according to the results (more than 40% and around 20%, respectively), which may possibly cause by the diverse encapsulation efficiency of the two drugs. The minor releasing rate of Ce6 demonstrated that it could be effectively protected in PLGA so that substantial ROS generation through LIFU stimulation could be reached to get a more convinced SDT efficiency. Another reason for this difference may be due to the different solvents of the two drugs during the preparation process. Specifically, DTX and PLGA were directly dissolved in dichloromethane, while Ce6 needed to be first dissolved in methanol and then added dropwise to dichloromethane, due to its poor solubility in the latter [53]. Since drug release in nanoparticles is directly related to the effectiveness of subsequent combination therapy, the assessment of drug-releasing rate as well as ROS generation result has mutually proved this point. Current researches have suggested that using chemotherapy alone may not significantly reverse tumor progression [41]. In this study, the nanoplatform we designed demonstrated strong evidence that compared with the single employment of chemotherapy, the synergistic treatment has remarkably elevated therapeutic efficiency both in vitro and in vivo. Since LIFU stimulation optimized the therapeutic strategy, an increased cell apoptosis rate was remarkably elevated. It is worth noting that due to this synergistic strategy, lung metastasis could be significantly inhibited both at tumor cell level and inoculated mice model, which is consistent with previous reports [16, 52].

## Conclusion

In conclusion, the safe and stable CPDP NPs we designed and prepared plus LIFU irradiation could remarkably eliminate breast tumor progression and its lung metastasis. With an enhanced imaging capability, this nanoplatform was also considered to be a promising contrast-enhanced agent in clinical. Hence, the novel synergistic strategy combined with LIFU might be considered as an effective treating application to reverse the poor outcome of metastatic breast cancer.

## Abbreviations

LIFU: Low-intensity focused ultrasound
SDT: Sonodynamic therapy
PFP: Perfluoropentane
EPR: Enhanced permeability and retention effect
DCFH-DA: Dichlorodihydrofluorescein diacetate
PI: Propidium iodide
TEM: Transmission electron microscope
ROS: Reactive oxygen species
Ce6: Chlorin e6
DTX: Docetaxel
FBS: Fetal bovine serum
CCK-8: Cell Counting Kit-8
CPDP NPs: Ce6/PFP/DTX/PLGA nanoparticles
PDP NPs: PFP/DTX/PLGA nanoparticles

## References

[1] Bray F (2018). Global cancer statistics 2018: GLOBOCAN estimates of incidence and mortality worldwide for 36 cancers in 185 countries. CA Cancer J Clin.

[2] Redig AJ (2013). Breast cancer as a systemic disease: a view of metastasis. J Intern Med.

[3] Li Z (2016). Emerging therapeutic targets in metastatic progression: a focus on breast cancer. Pharmacol Ther.

[4] Sutton TL, Alexander S, Gardiner SK, Nathalie J, Garreau JR (2020). Time to surgery following neoadjuvant chemotherapy for breast cancer impacts residual cancer burden, recurrence, and survival. J Surg Oncol.

[5] Fiocchetti M (2020). Extracellular neuroglobin as a stress-induced factor activating pre-adaptation mechanisms against oxidative stress and chemotherapy-induced cell death in breast cancer. Cancers (Basel).

[6] Varshosaz J (2015). Folated synperonic-cholesteryl hemisuccinate polymeric micelles for the targeted delivery of docetaxel in melanoma. Biomed Res Int.

[7] Wang M (2020). Development and evaluation of docetaxel-phospholipid complex loaded self-microemulsifying drug delivery system: optimization and in vitro/ex vivo studies. Pharmaceutics.

[8] Alshaker H (2017). New FTY720-docetaxel nanoparticle therapy overcomes FTY720-induced lymphopenia and inhibits metastatic breast tumour growth. Breast Cancer Res Treat.

[9] Xu M (2021). Sonodynamic therapy-derived multimodal synergistic cancer therapy. Cancer Lett.

[10] Liang S (2020). Recent advances in nanomaterial-assisted combinational sonodynamic cancer therapy. Adv Mater.

[11] Bhattacharya J, Chidambaram R, Qureshi T (2018). Ultrasonic-assisted synthesis of graphene oxide—fungal hyphae: an efficient and reclaimable adsorbent for chromium(VI) removal from aqueous solution. Ultrason Sonochem.

[12] An J (2020). ROS-augmented and tumor-microenvironment responsive biodegradable nanoplatform for enhancing chemo-sonodynamic therapy. Biomaterials.

[13] Jingxue W, Huang Ju, Weichen Z, Jiawen Z, Qi P, Liang Z, Zhigang W, Pan Li, Rui Li (2021). Hypoxia modulation by dual-drug nanoparticles for enhanced synergistic sonodynamic and starvation therapy. J Nanobiotechnology.

[14] Son S (2020). Multifunctional sonosensitizers in sonodynamic cancer therapy. Chem Soc Rev.

[15] Li Z (2020). Ce6-Conjugated and polydopamine-coated gold nanostars with enhanced photoacoustic imaging and photothermal/photodynamic therapy to inhibit lung metastasis of breast cancer. Nanoscale.

[16] Zhao H (2020). Biomimetic decoy inhibits tumor growth and lung metastasis by reversing the drawbacks of sonodynamic therapy. Adv Healthc Mater.

[17] Samuel Melvin S (2018). A GO-CS@MOF [Zn(BDC)(DMF)] material for the adsorption of chromium(VI) ions from aqueous solution. Compos B Eng.

[18] Samuel Melvin S, Sheriff SS, Jayanta B (2018). Adsorption of Pb(II) from aqueous solution using a magnetic chitosan/graphene oxide composite and its toxicity studies. Int J Biol Macromol.

[19] Samuel Melvin S, Jayanta B, Sankalp R (2019). Efficient removal of Chromium (VI) from aqueous solution using chitosan grafted graphene oxide (CS-GO) nanocomposite. Int J Biol Macromol.

[20] Samuel Melvin S, Swati S, Venkateshkannan (2020). Immobilization of Cu(btc) on graphene oxide-chitosan hybrid composite for the adsorption and photocatalytic degradation of methylene blue. J Photochem Photobiol B.

[21] Samuel Melvin S, Selvarajan E (2020). Synthesized β-cyclodextrin modified graphene oxide (β-CD-GO) composite for adsorption of cadmium and their toxicity profile in cervical cancer (HeLa) cell lines. Process Biochem.

[22] Samuel MS, Jose S, Selvarajan E (2020). (2020) Biosynthesized silver nanoparticles using Bacillus amyloliquefaciens; Application for cytotoxicity effect on A549 cell line and photocatalytic degradation of p-nitrophenol. J Photochem Photobiol B.

[23] Datta S, Veena R, Samuel MS (2020). Immobilization of laccases and applications for the detection and remediation of pollutants: a review. Environ Chem Lett.

[24] Alice AME, Melvin SS, Chidambaram R (2016). Application of rice husk nanosorbents containing 2,4-dichlorophenoxyacetic acid herbicide to control weeds and reduce leaching from soil. J Taiwan Inst Chem Eng.

[25] Samuel Melvin S, Jayanta B, Parthiban C (2018). Ultrasound-assisted synthesis of metal organic framework for the photocatalytic reduction of 4-nitrophenol under direct sunlight. Ultrason Sonochem.

[26] Abigail M, Samuel SM, Ramalingam C (2015). Addressing the environmental impacts of butachlor and the available remediation strategies: a systematic review. Int J Environ Sci Technol.

[27] Dong-Yang Z, Yue Z, Hang Z, Gang-Gang Y, Cai-Ping T, Liang He, Liang-Nian Ji, Zong-Wan M (2018). Folate receptor-targeted theranostic IrS nanoparticles for multimodal imaging-guided combined chemo-photothermal therapy. Nanoscale.

[28] Wang Y (2018). Multifunctional Cargo-free nanomedicine for cancer therapy. Int J Mol Sci.

[29] Yang M (2018). A modular coassembly approach to all-in-one multifunctional nanoplatform for synergistic codelivery of doxorubicin and curcumin. Nanomaterials (Basel).

[30] Yang C (2020). Dual ultrasound-activatable nanodroplets for highly-penetrative and efficient ovarian cancer theranostics. J Mater Chem B.

[31] Zhang L (2018). Size-modulable nanoprobe for high-performance ultrasound imaging and drug delivery against cancer. ACS Nano.

[32] Xu J (2011). Sonodynamic action of pyropheophorbide-a methyl ester induces mitochondrial damage in liver cancer cells. Ultrasonics.

[33] Shen X (2020). PLGA-based drug delivery systems for remotely triggered cancer therapeutic and diagnostic applications. Front Bioeng Biotechnol.

[34] Zhao Z (2017). A nanoparticle-based nicotine vaccine and the influence of particle size on its immunogenicity and efficacy. Nanomedicine.

[35] Shiva SA, Shahram MD (2020). Drug delivery of Amphotericin B through core–shell composite based on PLGA/Ag/FeO: in vitro test. Appl Biochem Biotechnol.

[36] Chen X (2019). A core-shell structure QRu-PLGA-RES-DS NP nanocomposite with photothermal response-induced M2 macrophage polarization for rheumatoid arthritis therapy. Nanoscale.

[37] Wang Y (2019). Biomimetic nanotherapies: red blood cell based core–shell structured nanocomplexes for atherosclerosis management. Adv Sci (Weinh).

[38] Mangrio FA, Dwivedi P, Han S (2017). Characteristics of Artemether-loaded poly(lactic-*co*-glycolic) acid microparticles fabricated by coaxial electrospray: validation of enhanced encapsulation efficiency and bioavailability. Mol Pharm.

[39] Cao Y (2018). Drug release from phase-changeable nanodroplets triggered by low-intensity focused ultrasound. Theranostics.

[40] García-Pinel B (2019). Lipid-based nanoparticles: application and recent advances in cancer treatment. Nanomaterials (Basel).

[41] Xiang Q (2021). Increased photodynamic therapy sensitization in tumors using a nitric oxide-based nanoplatform with ATP-production blocking capability. Theranostics.

[42] Yang M (2020). Fabrication of doxorubicin-gated mesoporous polydopamine nanoplatforms for multimode imaging-guided synergistic chemophotothermal therapy of tumors. Drug Deliv.

[43] Liu J (2018). Low-intensity focused ultrasound (LIFU)-activated nanodroplets as a theranostic agent for noninvasive cancer molecular imaging and drug delivery. Biomater Sci.

[44] Zhang L (2019). Mitochondria-targeted and ultrasound-activated nanodroplets for enhanced deep-penetration sonodynamic cancer therapy. ACS Appl Mater Interfaces.

[45] Pan X (2020). MOF-derived double-layer hollow nanoparticles with oxygen generation ability for multimodal imaging-guided sonodynamic therapy. Angew Chem Int Ed Engl.

[46] Truffi M (2019). Nano-strategies to target breast cancer-associated fibroblasts: rearranging the tumor microenvironment to achieve antitumor efficacy. Int J Mol Sci.

[47] Xie H (2017). Tumor microenvironment: driving forces and potential therapeutic targets for breast cancer metastasis. Chin J Cancer.

[48] Yiran L, Hanwen Z, Xiaojin S (2020). Metastatic heterogeneity of breast cancer: molecular mechanism and potential therapeutic targets. Semin Cancer Biol.

[49] Zheng X (2019). Biodegradable natural product-based nanoparticles for near-infrared fluorescence imaging-guided sonodynamic therapy. ACS Appl Mater Interfaces.

[50] Manaspon C (2017). Increasing distribution of drugs released from in situ forming PLGA implants using therapeutic ultrasound. Ann Biomed Eng.

[51] Sindeeva O (2018). Effect of a controlled release of epinephrine hydrochloride from PLGA microchamber array: in vivo studies. ACS Appl Mater Interfaces.

[52] Zhang D (2020). Ultrasound-driven biomimetic nanosystem suppresses tumor growth and metastasis through sonodynamic therapy, CO therapy, and indoleamine 2,3-dioxygenase inhibition. ACS Nano.

[53] Shirui M, Yi S, Luk Li, Jing Xu, Andreas S, Thomas K (2008). Effects of process and formulation parameters on characteristics and internal morphology of poly(d, l-lactide-co-glycolide) microspheres formed by the solvent evaporation method. Eur J Pharm Biopharm.

